# Effects of central administration of the human Tau protein on the Bdnf, Trkb, p75, Mapt, Bax and Bcl-2 genes expression in the mouse brain

**DOI:** 10.18699/VJGB-23-41

**Published:** 2023-07

**Authors:** A.S. Oreshko, A.Ya. Rodnyy, D.V. Bazovkina, V.S. Naumenko

**Affiliations:** Institute of Cytology and Genetics of the Siberian Branch of the Russian Academy of Sciences, Novosibirsk, Russia; Institute of Cytology and Genetics of the Siberian Branch of the Russian Academy of Sciences, Novosibirsk, Russia; Institute of Cytology and Genetics of the Siberian Branch of the Russian Academy of Sciences, Novosibirsk, Russia; Institute of Cytology and Genetics of the Siberian Branch of the Russian Academy of Sciences, Novosibirsk, Russia

**Keywords:** Alzheimer’s disease, Tau protein, Bdnf, neurogenesis, apoptosis, mice, болезнь Альцгеймера, Tau-белок, Bdnf, нейрогенез, апоптоз, мыши

## Abstract

Alzheimer’s disease is the most common form of dementia, affecting millions of people worldwide. Despite intensive work by many researchers, the mechanisms underlying Alzheimer’s disease development have not yet been elucidated. Recently, more studies have been directed to the investigation of the processes leading to the formation of neurofibrillary tangles consisting of hyperphosphorylated microtubule-associated Tau proteins. Pathological aggregation of this protein leads to the development of neurodegeneration associated with impaired neurogenesis and apoptosis. In the present study, the effects of central administration of aggregating human Tau protein on the expression of the Bdnf, Ntrk2, Ngfr, Mapt, Bax and Bcl-2 genes in the brain of C57Bl/6J mice were explored. It was found that five days after administration of the protein into the fourth lateral ventricle, significant changes occurred in the expression of the genes involved in apoptosis and neurogenesis regulation, e. g., a notable decrease in the mRNA level of the gene encoding the most important neurotrophic factor BDNF (brain-derived neurotrophic factor) was observed in the frontal cortex which could play an important role in neurodegeneration caused by pathological Tau protein aggregation. Central administration of the Tau protein did not affect the expression of the Ntrk2, Ngfr, Mapt, Bax and Bcl-2 genes in the frontal cortex and hippocampus. Concurrently, a significant decrease in the expression of the Mapt gene encoding endogenous mouse Tau protein was found in the cerebellum. However, no changes in the level or phosphorylation of the endogenous Tau protein were observed. Thus, central administration of aggregating human Tau protein decreases the expression of the Bdnf gene in the frontal cortex and the Mapt gene encoding endogenous mouse Tau protein in the cerebellum of C57Bl/6J mice.

## Abstract (ru)

Болезнь Альцгеймера – это наиболее распространенная форма деменции, вызывающая прогрессирующую утрату когнитивных способностей и поражающая миллионы людей во всем мире. Несмотря на интенсивную работу множества исследовательских групп, механизмы, лежащие в основе развития болезни Альцгеймера, до сих пор не выяснены. В последнее время все больше усилий направлено на изучение механизмов, приводящих к формированию внутриклеточных нейрофибриллярных клубков, состоящих из гиперфосфорилированного Tau-белка, ассоциированного с микротрубочками. Патологическая агрегация Tau-белка, как известно, приводит к развитию нейродегенерации, связанной с нарушением нейрогенеза и апоптоза. В данном исследовании мы рассмотрели эффекты центрального введения агрегирующего Tau-белка человека на паттерны экспрессии генов Bdnf, Ntrk2, Ngfr, Mapt, Bax и Bcl-2 в мозге мышей линии C57Bl/6J. Обнаружено, что через пять дней после введения Tau-белка человека в левый боковой желудочек мозга мыши происходят существенные изменения в паттернах экспрессии генов, принимающих участие в регуляции апоптоза и нейрогенеза. Так, было показано значительное снижение уровня мРНК гена Bdnf, кодирующего важнейший нейротрофический фактор мозга (brain-derived neurotrophic factor), во фронтальной коре мозга мышей экспериментальной группы, что может играть важную роль в нейродегенерации, вызываемой патологической агрегацией Tau-белка. В то же время центральное введение Tau-белка человека не повлияло на экспрессию генов Ntrk2, Ngfr, Mapt, Bax и Bcl-2 во фронтальной коре и гиппокампе мышей. При этом в мозжечке было обнаружено существенное снижение экспрессии гена Mapt, кодирующего эндогенный Tau-белок мыши. Однако изменений в уровне белка и фосфорилировании эндогенного Tau-белка в исследованных структурах мозга не выявлено. Таким образом, центральное введение агрегирующего Tau-белка человека приводит к снижению экспрессии гена Bdnf во фронтальной коре и гена эндогенного Tau-белка (Mapt) в мозжечке мышей линии С57Bl/6J.

## Introduction

Alzheimer’s disease (AD) is the most common cause of dementia with a prevalence of 24 million and an incidence of up to 5 million cases per year (Ferri et al., 2005). Russia is among the nine countries with the highest number of people suffering from AD (Prince et al., 2013; Collaborators, 2019). The annual death rate from AD and other forms of dementia in Russia in 2016 reached 35.7 per 100,000 inhabitants (https:// www.who.int/healthinfo/global_burden_disease/estimates/ en/), however, since many cases of AD remain unreported, this number is likely to be greatly underestimated

AD is characterized by two main histopathological features: (1) extracellular amyloid plaques formed by insoluble aggregates of hydrophobic beta-amyloid peptides and (2) intracellular neurofibrillary tangles composed of hyperphosphorylated microtubule-associated Tau proteins. The accumulation of these two major types of aggregates leads to irreversible neurodegeneration that slowly spreads throughout the brain and causes progressive memory loss, cognitive decline, severe dementia and, finally, death (Breijyeh, Karaman, 2020). Although many generations of researchers have tried to unravel the mechanisms underlying this disease, they are still far from being fully understood. In recent years, the attention of an increasing number of scientists has been directed to studying the mechanisms leading to Tau pathology development

Tau protein is a member of the microtubule-associated protein (MAP) family. Physiologically, this protein is involved in the formation and stabilization of microtubules in neurons and in the regulation of axonal transport and axon growth (Avila et al., 2004). The protein’s main function is the regulation of tubulin polymerization, but it has also been shown to have DNA/RNA protection and signaling functions as well as to play a role in transcription regulation (Mandelkow E.M., Mandelkow E., 2012; Tapia-Rojas et al., 2019; Wegmann et al., 2021; Giovannini et al., 2022). Under pathological conditions, the accumulation of the protein’s insoluble aggregates leads to the development of neurodegeneration, which is obviously leads to deteriorated neurogenesis and apoptosis, e. g., it has been shown that the level of Tau protein expression negatively correlates with the expression of BDNF (Wei et al., 2022) playing an important role in neuron development and support (Lu, Figurov, 1997; Benarroch, 2015; Gulyaeva, 2017). A similar correlation has been demonstrated for pathological Tau protein hyperphosphorylation (Yuan et al., 2022). Increased Tau protein expression also leads to a decrease in the BDNF level of blood plasma (Alvarez et al., 2022). Various actions that increase BDNF expression suppress the expression and pathological hyperphosphorylation of Tau protein (Li et al., 2022; Lin et al., 2022). Medications, including those of plant origin, that improve the performance of cognitive tasks in various models, reduce the expression of Tau protein and the proapoptotic BAX protein gene, which is accompanied by an increase in the expression of the antiapoptotic BCL- 2 protein (Huang et al., 2022; Tu et al., 2022; Zhang et al., 2022), an increase in BDNF expression, as well as an increase in the expression and phosphorylation of the TrkB receptors mediating the positive effects of BDNF (Zhao et al., 2021; Liu et al., 2022; Nandini et al., 2022; Saikia et al., 2022; Wang et al., 2022). TrkB receptor activation has led to a decrease in Tau protein phosphorylation both in vitro, in cell culture, and in animal models (Chiang et al., 2021; Liao et al., 2021; Gonzalez et al., 2022).

The association between the nonspecific p75 receptor mediating the proapoptotic effects of the BDNF precursor (Guo et al., 2016; Hashimoto, 2016) and Tau pathology is less clear. Some studies have demonstrated that the adverse effects of aging and inflammation may be mediated at least partially by increased expression of the p75 receptor (Xie et al., 2021). At the same time, p75 receptor blockade suppresses proNGF (nerve growth factor precursor)-induced Tau protein phosphorylation (Shen et al., 2018). LM11A-31, a p75 re-ceptor antagonist, also suppresses hyperphosphorylation and pathological aggregation of Tau protein in a mouse AD model (Yang et al., 2020).

However, it remains unclear what effect exerts introduction of aggregating human Tau proteins on the expression patterns of the genes involved in the processes of neurogenesis and apoptosis in the mouse brain. The aim of this study was to investigate the possibility of using standard C57Bl/6J mice after administration of aggregating human Tau protein into the left lateral ventricle as a model for studying Tau pathology mechanisms. In particular, we planned to evaluate the effects of the Tau protein administration on the expression patterns of the Bdnf, Ntrk2 (encodes the TrkB receptor), Ngfr (encodes the p75 receptor), Mapt (encodes endogenous Tau), Bax, and Bcl-2 genes in the mouse brain as well as on the level and phosphorylation of endogenous mouse Tau protein.

## Materials and methods

Experimental animals. The investigation was carried out on C57Bl/6J inbred male mice of 10–12 weeks old weighing 27 ± 0.3 g at Center for Genetic Resources of Laboratory Animals of the Institute of Cytology and Genetics, Siberian Branch of the Russian Academy of Sciences (RFMEFI62119X0023).

In all experimental series, the animals were kept under the standard conditions of the vivarium that included artificial 14-hour lighting, 60 % humidity, temperature of 23 °C and a free access to balanced food and water. All the procedures involving experimental animals were performed in accordance with the international rules for the treatment of animals (Directive 2010/63/EU) and Order of the Ministry of Health of the Russian Federation on Approval of the Rules of Good Laboratory Practice of 04/01/2016 No. 199n (registered on 08/15/2016 No. 43232).

Intraventricular administration of Tau protein. The human Tau protein was synthesized at Convergence Research Center for Diagnosis, Treatment and Care System of Dementia, Brain Science Institute, Korea Institute of Science and Technology (KIST) and kindly provided by the Director of the Institute, Dr. Yun Kyung Kim.

The protein was diluted in DMSO to a concentration of 2 mg/ml and then diluted with saline to a concentration of 0.2 μg/μl to be microinjected into the left lateral ventricle of the mice’s brain (i.c.v.), AP: –0.5, L: –1.6 mm, DV: 2 mm (Slotnick, Leonard, 1975) under stereotaxic control (TSE, Germany). Before the injection, the mice had been narcotized for 20–30 sec with diethyl ether (Kondaurova et al., 2012). Mice in the control group received an injection of the solvent of the same composition. The volume of centrally injected fluids was 5 μl. Three days after the injection, the animals were placed in individual cages to remove group effects. After 46–48 hours, the mice were decapitated, their frontal cortex, hippocampus and cerebellum (as a control brain structure that is less involved in the implementation of the hyperphosphorylation and aggregation effects of Tau protein on cognition) were frozen in liquid nitrogen and stored at –80 °C prior total RNA isolation and western blotting.

RT-PCR. Total RNA was isolated using the TRIzol reagent (ThermoScientific, USA) and 1 μg of mRNA was used for synthesizing cDNA with random hexanucleotide primer. PCR was performed as in our previous studies (Naumenko et al., 2013a, b; Kondaurova et al., 2020). Real-time quantitative PCR was performed using the primers described in the Table. Gene expression was presented as the number of cDNA copies relative to 100 copies of Polr2a cDNA (Kulikov et al., 2005; Naumenko, Kulikov, 2006; Naumenko et al., 2008).

**Table 1.**
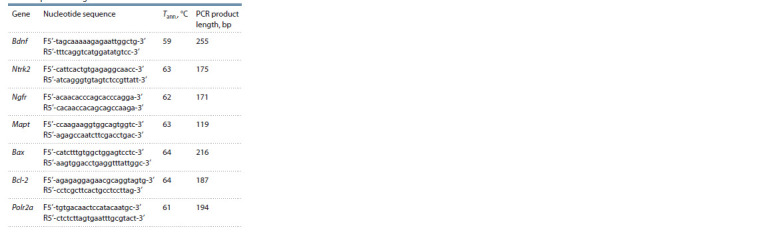
The primer sequences, annealing temperatures, and PCR product lengths

Western blotting. The analysis was performed in the way it had been done in our previous works (Ilchibaeva et al., 2018; Popova et al., 2020). In brief, the total protein fraction was isolated from brain samples. The samples were then separated by 10 % SDS-PAGE and transferred to a nitrocellulose membrane. The membrane was then blocked with 5 % skimmed milk powder or 5 % BSA (for Phospho-Tau Thr18) for 1 hour and then incubated with primary antibodies to Tau proteins (5A6, 1:1000, DSHB, USA) and GAPDH (CAB932Hu01, 1:2500, Cloud-Clone Corp., USA) in 5 % milk powder with TBS-T or in 5 % FBS with TBS-T for Phospho-Tau Thr181 (AT270, 1:1000, Thermo Fisher Scientific, USA) for 16 hours at 4 °C. For protein detection, the membranes were incubated with horseradish peroxidase conjugated with secondary antibodies (anti-mouse Ig ab6728, 1:20000, Invitrogen, USA, Abcam, UK) in 5 % FBS with TBS-T for 1 hour at room temperature. Protein bands were visualized in a C-DiGit chemiluminescent blot scanner (LI-COR, USA) using the Clarity Western ECL substrate (Bio-Rad., USA). The bands were quantified using the Image Studio software (LICOR, USA). Target protein levels were normalized to that of GAPDH expression, which is constitutive of brain cells, and presented as a percentage of control animals. The number of analyzed samples was n ≥ 8.

Statistical analysis. The results were presented as m ± SEM, where m is the mean and SEM is the standard error of the mean. The samples were compared using a oneway ANOVA. The differences were considered significant at p < 0.05. The normality of the variances was tested using the Kolmogorov–Smirnov and Shapiro–Wilk tests. Dixon’s test was applied to identify and exclude extreme deviations from the analysis.

## Results

**Fig. 1.**
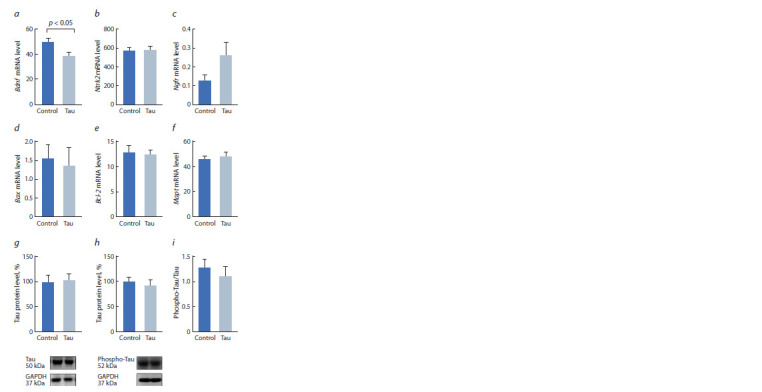
Effect of central administration of human Tau protein on the expression of Bdnf (a), Ntrk2 (b), Ngfr (c), Bax (d ) and Bcl-2 (e) genes, as well as on the Mapt gene mRNA level (f ), Tau protein (g), phosphorylated Tau protein (h) and the ratio of phosphorylated Tau protein to Tau protein (i ) in the frontal cortex of C57Bl/6J mice Here and in Fig 2 and 3: the gene expression is presented as the number of cDNA copies of the corresponding gene per 100 copies of Polr2a cDNA. The protein level is presented in relative units of the chemiluminescent signal and normalized to the level of GAPDH protein. n ≥ 8.

Central administration of the human Tau protein resulted in a change in Bdnf gene expression. A significant decrease in the expression of this gene was found in the frontal cortex of the mice of the experimental group (F1,13 = 7.2, p <0.05) (Fig. 1, a).

At the same time, no changes were found in the expression of genes encoding BDNF receptors (F1,14 = 0.08 for Ntrk2 and F1,14 = 1.9, p > 0.05 for Ngfr, see Fig. 1, b, c). Also, the Tau protein had no effect on the expression of the genes encoding proapoptotic factor BAX (F1,13 = 0.08) and antiapoptotic factor BCL-2 (F1,14 = 0.06, see Fig. 1, d, e). Tau protein administration did not lead to significant changes in the expression of endogenous Tau protein both at the mRNA (F1,14 = 0.2) and protein levels (F1,14 = 0.034, see Fig. 1, f, g). The phosphorylation of endogenous Tau protein also did not change (F1,14 = 0.273 for the phospho-Tau level and F1,14 = 0.393 for the phospho-Tau/Tau ratio, see Fig. 1, h, i).

**Fig. 2.**
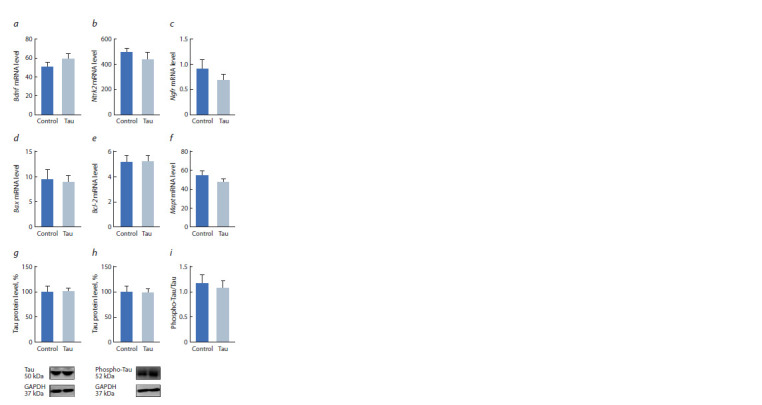
Effect of central administration of human Tau protein on the expression of Bdnf (a), Ntrk2 (b), Ngfr (c), Bax (d ) and Bcl-2 (e) genes, as well as on the Mapt gene mRNA level (f ), Tau protein (g), phosphorylated Tau protein (h) and the ratio of phosphorylated Tau protein to Tau protein (i ) in the hippocampus of C57Bl/6J mice.

In hippocampus, Tau protein administration did not cause any significant changes in the expression pattern of the studied genes (F1,13 = 1.2, p > 0.05 for Bdnf; F1,14 = 0.8 for Ntrk2; F1,13 =1.0, p > 0.05 for Ngfr; F1,12 = 0.004 for Bcl-2; F1,10 =0.1 for Bax) (Fig. 2, a–e).

Also, no significant changes were found in the expression of the endogenous Tau protein both at the mRNA (F1,14 = 1.3, p > 0.05) and protein levels (F1,14 = 0.508, see Fig. 2, f, g). The phosphorylation of endogenous Tau protein did not change as well (F1,14 = 0.012 for the phospho-Tau level; F1,13 = 0.015 for the phospho-Tau/Tau ratio, see Fig. 2, h, i).

**Fig. 3.**
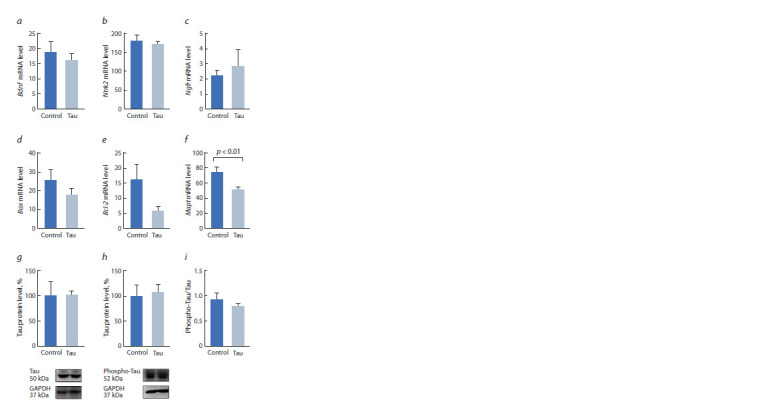
Effect of central administration of human Tau protein on the expression of Bdnf (a), Ntrk2 (b), Ngfr (c), Bax (d ) и Bcl-2 (e) genes, as well as on the Mapt gene mRNA level (f ), Tau protein (g), phosphorylated Tau protein (h) and the ratio of phosphorylated Tau protein to Tau protein (i ) in the cerebellum of C57Bl/6J mice.

In the cerebellum, the human Tau protein also did not affect the expression of Bdnf (F1,14 = 0.3), Ntrk2 (F1,14 = 0.5), Ngfr (F1,14 = 2.4, p > 0.05), and Bax (F1,11 = 1.4, p > 0.05) genes (Fig. 3, a–d ). At the same time, a trend towards a decrease in the anti-apoptotic Bcl-2 gene was found (F1,14 = 3.8753, p = 0.076, see Fig. 3, e). Interestingly, Tau protein administration had a significant effect on the expression of the gene encoding endogenous Tau protein in the cerebellum (F1,11 = 9.7, p > 0.01, see Fig. 3, f ). However, no significant changes were found in the level of endogenous Tau protein (F1,13 = 0.043) as well as in the level of its phosphorylation (F1,13 = 0.107 for the phospho-Tau level; F1,13 = 0.011 for the phospho-Tau/Tau ratio, see Fig. 3, g–i).

## Discussion

AD is one of the most common causes of dementia that affects millions of people. It also leads to a large burden for the health and care systems. Despite intensive research worldwide, AD mechanisms remain unclear and only symptomatic treatment is available to date

AD is characterized by the formation of two types of protein aggregates leading to the development of neurodegeneration. These are accumulations of extracellular amyloid plaques and intracellular neurofibrillary tangles, consisting of hyperphosphorylated microtubule-associated Tau proteins. Since longterm studies of the amyloid pathology have not brought the desired results, in recent years more and more research groups have directed their attention to investigating the mechanisms underlying the Tau pathology.

In this study, we investigated how central administration of human Tau protein in mice affects the expression patterns of the genes involved in the processes of neurogenesis and apoptosis, as well as the level and phosphorylation of endogenous Tau protein. It was shown that Tau protein administration into the lateral ventricle led to a significant decrease in the expression of the gene encoding BDNF in the frontal cortex. Considering the critical role of this factor in neurons development and support (Lu, Figurov, 1997; Benarroch, 2015; Gulyaeva, 2017), it can be assumed that a decrease in Bdnf gene expression can lead to the development of neurodegeneration. Here, it has to be emphasized that the most pronounced neurodegenerative changes, as well as cell function changes in AD, have been observed precisely in the frontal cortex and hippocampus (Guevara et al., 2022; Lee et al., 2022). This is due, among other things, to the enhanced accumulation of Tau protein aggregates in these brain structures (Shimada et al., 2020) and the role of these brain structures in cognitive function regulation

It is noteworthy that Tau protein administration also had its effect on the cerebellum that is not that much involved in cognitive processes. Our study demonstrated that the expression of the Mapt gene encoding the endogenous Tau protein was reduced in the cerebellum of the mice of the experimental group. In part, these results are consistent with the data on accumulation of Tau protein aggregates in this brain structure (Guevara et al., 2022). However, the detected changes in the Tau protein mRNA level in the cerebellum did not lead to significant changes in the level of endogenous Tau protein and its phosphorylation. Also, no changes were observed in the levels of mRNA, protein, and phosphorylation of the endogenous Tau protein in all structures studied. The central administration of Tau protein did not significantly affect the expression of other studied genes.

In general, our data agree with those on a negative correlation between Tau protein expression (Wei et al., 2022) and hyperphosphorylation (Yuan et al., 2022) with BDNF expression. However, despite the well-documented relationship between Tau protein expression and that of pro- and anti-apoptotic genes (Huang et al., 2022; Tu et al., 2022; Zhang et al., 2022), as well as the gene encoding the TrkB receptor (Zhao et al., 2021; Liu et al., 2022; Nandini et al., 2022; Saikia et al., 2022; Wang et al., 2022), the administration of Tau protein did not affect the expression patterns of these genes. An assumption can be made that the exogenous Tau protein introduced into the intercellular space penetrates poorly inside the neurons or is quickly catabolized there, not having the time to initiate a process of cells degeneration. Probably, for a more thorough investigation of the effects of Tau protein aggregation on the brain function, it is necessary to ensure endogenous expression of the pathologically phosphorylated Tau proteins in neurons

## Conclusion

The results obtained in this study indicate that central administration of human Tau protein to C57Bl/6J mice has a very weak effect on the expression of the investigated genes involved in neurogenesis and apoptosis. Nevertheless, Tau protein administration has led to a decrease in the expression of Bdnf gene in the frontal cortex and endogenous Tau proteinencoding gene in the cerebellum of C57Bl/6J mice without affecting the level and phosphorylation of endogenous Tau protein in the studied brain structures.

## Conflict of interest

The authors declare no conflict of interest.
